# Is there a Role for Preoperative Infusion or Intraoperative Cholangiography?

**DOI:** 10.1155/1997/30872

**Published:** 1997

**Authors:** W. Y. Lau, Arthur K. C. Li

**Affiliations:** Department of Surgery The Chinese University of Hong Kong Prince of Wales Hospital Shatin Hong Kong

## Abstract

*Background*: There has been a resurgence of interest in recent years in preoperative infusion cholangiography (PIC). The role of routine PIC compared to routine intraoperative cholangiography (IOC) has not been clearly defined.

*Study design*: In our department between 1985 and 1991, 1,042 of 1,576 consecutive patients with biliary calculous disease had elective cholecystectomy: 694 patients were prospectively scheduled for PIC, and 348 patients were randomly allocated to IOC. The patients in the PIC and IOC groups were similar with regard to age, history of biliopancreatic complications, and laboratory findings. The cost of PIC in Sweden is nearly five times greater than the cost of IOC.

*Results*: Satisfactory opacification of the biliary system was obtained in 90.1 and 96.8 percent of patients who underwent PIC and IOC, respectively. Preoperative infusion cholangiography required support by IOC in 19.5 percent of patients. There  were no statistically significant differences between the PIC and IOC groups with regard to the incidence (7 percent in both groups) of or positive predictive value (68 and 80 percent, respectively) for bile duct stones, rate of retained stones (6 and 20 percent, respectively), intraoperative (5.6 and 6.3 percent, respectively) or postoperative (13.3 and 15.9 percent, respectively) morbidity, or incidence of bile duct anomalies (0.9 and 0.3 percent, respectively). Median operative time was longer in .patients with (95 minutes) compared to those without (75 minutes) IOC (*p*<0.001). More postoperative complications occurred after bile duct exploration (26 of 75 patients) compared to cholecystectomy alone (114 of 917 patients, *p*<0.001). The 30-day mortality was zero. Minor bile duct injuries occurred in two patients (0.2 percent) at cholecystectomy, (one with and one without bile duct exploration). In no patient was the cholangiographic finding of a biliary anomaly crucial for the safe execution of cholecystectomy.

*Conclusions*: In our study, PIC and IOC were comparable, but routine use of either method did not promote the safety of cholecystectomy and thus their routine use is not warranted. The shorter operative time and preoperative identification of common bile duct (CBD) stones provided by PIC might favor this examination when applied selectively  in patients with increased risk of having CBD stones. However, this potential advantage is offset by the need for PIC to be supported by IOC in approximately 20 percent of patients. Also, the cost of PIC is greater than the cost of IOC.


### Is there a Role for Preoperative Infusion or Intraoperative Cholangiography?

### ABSTRACT

*Hammarström, E-K., Holmin, T., Stridbeck, H. and Ihse, I. (1996) Routine preoperative infusion cholangiography versus intraoperative cholangiography at elective cholecystectomy: A prospective study in 995 patients, Jounral of the American College of Surgeons; 182, 408–416.*

***Background:*** **There has been a resurgence of interest in recent years in preoperative infusion cholangiography (PIC). The role of routine PIC compared to routine intraoperative cholangiography (IOC) has not been clearly defined.**
***Study design:*** **In our department between 1985 and 1991, 1,042 of 1,576 consecutive patients with biliary calculous disease had elective cholecystectomy: 694 patients were prospectively scheduled for PIC, and 348 patients were randomly allocated to IOC. The patients in the PIC and IOC groups were similar with regard to age, history of biliopancreatic complications, and laboratory findings. The cost of PIC in Sweden is nearly five times greater than the cost of IOC.**
***Results:*** **Satisfactory opacification of the biliary system was obtained in 90.1 and 96.8 percent of patients who underwent PIC and IOC, respectively. Preoperative infusion cholangiography required support by IOC in 19.5 percent of patients. There were no statistically significant differences between the PIC and IOC groups with regard to the incidence (7 percent in both groups) of or positive predictive value (68 and 80 percent, respectively) for bile duct stones, rate of retained stones (6 and 20 percent, respectively), intraoperative (5.6 and 6.3 percent, respectively) or postoperative (13.3 and 15.9 percent, respectively) morbidity, or incidence of bile duct anomalies (0.9 and 0.3 percent, respectively). Median operative time was longer in patients with (95 minutes) compared to those without (75 minutes) IOC ($p$<0.001). More postoperative complications occurred after bile duct exploration (26 of 75 patients) compared to cholecystectomy alone (114 of 917 patients, $p$<0.001). The 30-day mortality was zero. Minor bile duct injuries occurred in two patients (0.2 percent) at cholecystectomy, (one with and one without bile duct exploration). In no patient was the cholangiographic finding of a biliary anomaly crucial for the safe execution of cholecystectomy.**
***Conclusions:*** **In our study, PIC and IOC were comparable, but routine use of either method did not promote the safety of cholecystectomy and thus their routine use is not warranted. The shorter operative time and preoperative identification of common bile duct (CBD) stones provided by PIC might favor this examination when applied selectively in patients with increased risk of having CBD stones. However, this potential advantage is offset by the need for PIC to be supported by IOC in approximately 20 percent of patients. Also, the cost of PIC is greater than the cost of IOC, *J. Am. Coll. Surg.*, 1996, 182, 408–416.**

*Keywords:* Infusion cholangiography, intraoperative cholangiography, cholangiography, cholecystectomy, bile duct resection

### PAPER DISCUSSION

In the era of open cholecystectomy, whether intraoperative cholangiography (IOC) for gallstone disease should be performed routinely or selectively has been controversial. Advocates of its routine use argue that IOC detects asymptomatic common bile duct (CBD) calculi, ductal anomalies and tumours of the bile duct [1–6]. The incidence of a positive yield with IOC, however, is low. Despite using the classical criteria of a dilated CBD, multiple gallbladder stones, history of pancreatitis and jaundice as indicators of high risk for choledocholithiasis, the actual CBD stone rate in about 5000 patients was only 5% [7]. In another study involving 280 patients with no criteria indicating possible choledocholithiasis who underwent open cholecystectomy and were randomised to IOC or no IOC, no patients from either group had retained CBD calculi over a 12 month follow-up period. However, the IOC was falsely positive in 3 patients (2.1%) and 2 were subjected to unnecessary CBD exploration [8]. Although CBD exploration is safe, Doyle *et al.* reported the mortality of patients with CBD exploration was 2.4 times greater than that for cholecystectomy alone [2].

The routine use of preoperative infusion cholangiography (PIC) as compared with routine IOC was studied prospectively by Hammerstrom and colleagues at elective open cholecystectomy. PIC failed to opacify or adequately opacify the bile ducts in 9.9% of patients. Furthermore IOC was necessary in 19.5% of patients who had had PIC performed [9]. Faced with the financial stringency of a cost conscious health system, the much higher cost of performing PIC (5 times the cost of IOC) cannot be justified. The estimated hospital charge for each IOC is US $641 [10]. In 1987, 600,000 cholecystectomies were performed in the United States [11]. Even if the number of cholecystectomies performed were to remain static until 1996, the cost of routine IOC alone in the United States would be a staggering US $384.6 millions for 1996!

Both intravenous cholangiography and ultrasonography have the advantage of providing preoperative knowledge of the bile duct status. Intravenous cholangiography can directly visualize the duct but the technique takes longer than ultrasonography to perform, uses radiation and has a low risk of allergic reaction which makes it less attractive than ultrasonography. Preoperative ultrasonography and liver function tests are widely used for assessing the bile duct for stones but these tests have the limitation of a poor positive predictive value of 14 – 58% for stones [12–17]. Bile duct stones can and do migrate through the sphincter of Oddi thus making any form of radiological and/or biochemical assessment of the bile duct valid only if performed close to the time to operation. Welbourn *et al.*, in a prospective study evaluating ultrasonography and liver function tests for preoperative assessment of stones within the bile duct, demonstrated that if the ultrasonography and/or liver function tests performed on the day before operation were normal then the negative predictive value for the absence of stones was at least 96% [18].

The use of intraoperative ultrasonography (IOUS) during open biliary tract surgery in screening for bile duct stones at the time of cholecystectomy is a more attractive option than IOC. In 666 IOUS and 401 IOC, IOUS was technically unsuccessful in only 1.2% of cases compared to 5.5% with IOC. The positive predictive value of IOUS (94.8%) was significantly better than that of IOC (71.7%) [19]. IOUS has a 94% accuracy in predicting the presence of bile duct stones if the size of gallstones present were $\leq$ 3 mm and the bile duct diameter was $\geq$ 7 mm [20]. An added advantage of IOUS is it takes less time to perform than IOC.

The advent of endoscopic retrograde cholangiopancreatography (ERCP) in 1970 and a diathermy sphincterotome in 1974 made endoscopic extraction of CBD stones a reality. With a success rate of 85–92% of clearance of CBD stones [21], preoperative ERCP combined with cholecystectomy was thought to be superior to open cholecystectomy and CBD exploration. However, this has not been substantiated in randomized trials [22–24]. In 9–29% of cases, the sheer size of the stone or the disproportion between the diameter of the distal CBD and the size of the stone precludes endoscopic removal [22, 24, 25] and these patients will still come to open CBD exploration.

Although the case for selective cholangiography in open cholecystectomy has been well made [8, 26], the birth and success of laparoscopic cholecystectomy has rekindled the controversy about routine or selective use of cholangiography in laparoscopic cholecystectomy. The experience gained from the era of open cholecystectomy cannot be directly extrapolated to laparoscopic cholecystectomy. There are proponents [27] and opponents [28] of routine cholangiography during laparoscopic cholecystectomy. Equally the question of whether ERCP should be performed before or after laparoscopic cholecystectomy in the presence of ductal stones remains conjectural. A recent decision analysis study by Erickson and Carlson concluded that postoperative ERCP for ductal stones discovered at laparoscopic cholecystectomy was more cost effective and produces less morbidity than preoperative ERCP and open CBD exploration (no comparison was performed on laparoscopic CBD exploration). When choledocholithiasis is likely, the result of selective preoperative ERCP is comparable to postoperative ERCP [29]. The issue has become even more complicated because CBD stones can now be dealt with by laparoscopic CBD exploration at the time of the laparoscopic cholecystectomy. Whether laparoscopic treatment of CBD stone has any advantages over ERCP and endoscopic stone retrieval remains unknown. It is only with proper randomised studies that definitive answers to all these questions can be provided in this era of laparoscopic surgery.

### *References*

[1] Cranley, B. and Logan, H. (1980). Exploration of the common bile duct – the relevance of the clinical picture and the importance of peroperative cholangiography, *Br. J. Surg.*, **67**, 869–872.

[2] Doyle, P. J., Ward-McQuaid, J. N. and McEwen Smith, A. (1982). The value of routine peroperative cholangiography – a report of 4000 cholecystectomies. *Br. J. Surg.*, **69**, 617–619.

[3] Faris, I., Thomson, J. P. S., Grundy, D. J. and LeQuesne L. P. (1975). Operative cholangiography: A reappraisal based on a review of 400 cholangiograms. *Br. J. Surg.*, **62**, 966–972.

[4] Holliday, H. J., Farringer, J. L., Terry, R. B. and Pickens, D. R. (1980). Operative cholangiography: Review of 7529 operations on the biliary tree in a community hospital. *Am. J. Surg.*, **139**, 379–382.

[5] Hermann, R. E. (1990). Surgery for acute and chronic cholecystitis. *Surg. Clin. North Am.*, **70**, 1263–1275.

[6] Shively, E. H., Wieman, T. J., Adams, A. L., Romines, R. B. and Garrison, R. N. (1990). Operative cholangiography. *Am. J. Surg.*, **159**, 380–384.

[7] Strasberg, S. M. and Soper, N. J. (1995). Management of choledocholithiasis in the laparoscopic era. *Gastroenterology*, **109**, 320–322.

[8] Hauer-Jensen, M., Karesen, R., Nygaard, K., Solheim, K. Amlie, E., Havig, O. and Viddal, K. O. (1986). Consequences of routine peroperative cholangiography during cholecystectomy for gallstone disease: A prospective, randomized study. *World, J. Surg.*, **10**, 996–1002.

[9] Hammarstron, L. E., Holmin, T., Stridbeck, H. and Ihse, I. (1996). Routine preoperative infusion cholangiography versus intraoperative cholangiography at elective cholecystectomy: A prospective study in 995 patients. *J. Am. Coll. Surg.*, **182**, 408–416.

[10] Orlando, R. III, and Russell, J. C. (1996). Managing gallbladder disease in a cost-effective manner. *Surg. Clin. North Am.*, **76**, 117–128.

[11] Bogokowsky, H., Slutzki, S., Zaidenstein, L., Halpern Z., Negri, M. and Abramsohn, R. (1987). Selective operative cholangiography. *Surg. Gynecol. Obstet.*, **164**, 124–126.

[12] Neuhaus, H., Feussner, H., Ungeheuer, A., Hoffmann, W., Siewert, J. R. and Classen, M. (1992). Prospective evaluation of the use of endoscopic retrograde cholangiography prior to laparoscopic cholecystectomy. *Endoscopy*, **24**, 745–749.

[13] O'Rourke, N. A., Askew, A. R., Cowen, A. E., Roberts, R. and Fielding, G. A. (1993). The role of ERCP and endoscopic sphincterotomy in the era of laparoscopic cholecystectomy. *Aust. N. Z. J. Surg.*, **63**, 3–7

[14] Leitman, I. M., Fisher, M. L., McKinley, M. J., Rothman, R., Ward, R. J., Reiner, D. S. and Tortolani, A. J. (1993). The evaluation and management of known or suspected stones of the common bile duct in the era of minimal access surgery. *Surgery Gynecol. Obstet.*, **176**, 527–533.

[15] Hawasli, A., Lloyd, L., Pozios, V. and Veneri, R. (1993). The role of endoscopic retrograde cholangio-pancreaticogram in laparoscopic cholecystectomy. *Am. Surg.*, **59**, 285–289.

[16] Graham, S. M., Flowers, J. L., Scott, T. R., Bailey, R. W., Scovill, W. A., Zucker, K. A. and Imbembo, A. L. (1993). Laparoscopic cholecystectomy and common bile duct stones. The utility of planned perioperative endoscopic retrograde cholangiography and sphincterotomy: Experience with 63 patients. *Ann. Surg.*, **218**, 61–67.

[17] Madhavan, K. K., Macintyre, I. M. C., Wilson, R. G., Saunders, J. H., Nixon, S. J. and Hamer-Hodges, D. W. (1995). Role of intraoperative cholangiography in laparoscopic cholecystectomy. *Br. J. Surg.*, **82**, 249–252.

[18] Welbourne, C. R. B., Haworth, J. M., Leaper, D. J. and Thompson, M. H. (1995). Prospective evaluation of ultrasonography and liver function tests for preoperative assessment of the bile duct. *Br. J. Surg.*, **82**, 1371–1373.

[19] Machi, J. and Sigel, B. (1996). Operative ultrasound in general surgery. *Am. J. Surg.*, **172**, 15–20.

[20] Grieg, J. D., John, T. G., Mahadevan, M. and Garden, O. J. (1994). Laparoscopic ultrasonography in the evaluation of the biliary tree during laparoscopic cholecystectomy. *Br. J. Surg.*, **81**, 1202–1206.

[21] Gordon, R. L. and Shapiro, H. A. (1990). Nonoperative management of bile duct stones. *Surg. Clin. North Am.*, **70**, 1313–1328.

[22] Neoptolemos, J. P., Carr-Locke, D. I. and Fossard, D. P. (1987). Prospective randomized study of preoperative endoscopic sphincterotomy versus surgery alone for common bile duct stones. *BMJ*, **294**, 470–474.

[23] Stain, S. C., Cohen, H., Tsuishoysha, M. and Donovan, A. J. (1991). Choledocholithiasis. Endoscopic sphincterotomy or common bit duct exploration. *Ann. Surg.*, **213**, 627–632.

[24] Stiegmann, G. V., Goff, J. S., Mansour, A., Pearlman, N., Reveille, R. M. and Norton, L. (1992). Precholecystectomy endoscopic cholangiography and stone removal is not superior to cholecystectomy, cholangiography, and common duct exploration. *Am. J. Surg.*, **163**, 227–230.

[25] Ponchon, T., Bory, R., Chavaillon, A. and Fouillet, Ph., (1989). Biliary lithiasis: Combined endoscopic and surgical treatment, *Endoscopy*, **21**, 15–18.

[26] Taylor, T. V. (1990). Editorial comment. *Am. J. Surg.* 159:385.

[27] Soper, N. J. and Brunt, L. M. (1994). The case for routine operative cholangiography during laparoscopic cholecystectomy. *Surg. Clin. North Am.*, **74**, 953–959.
[28] Clair, D. G. and Brooks, D. C. (1994). Laparoscopic cholangiography: The case for a selective approach. *Surg. Clin. North Am.*, **74**, 961–966.
[29] Erickson, R. A. and Carlson, B. (1995). The role of endoscopic retrograde cholangiopancreatography in patients with laparoscopic cholecystectomies *Gastroenterology*, **109**, 252–263.

W. Y. Lau
Arthur K. C. Li
Department of Surgery
The Chinese University of Hong Kong
Prince of Wales Hospital
Shatin
Hong Kong

